# Review of the Recent Literature on the Mode of Delivery for Singleton Vertex Preterm Babies

**DOI:** 10.1155/2011/186560

**Published:** 2011-07-24

**Authors:** Smriti Ray Chaudhuri Bhatta, Remon Keriakos

**Affiliations:** Obstetrics and Gynaecology, Sheffield Teaching Hospitals, Sheffield S10 2SF, UK

## Abstract

Choosing the safest method of delivery and preventing preterm labour are obstetric challenges in reducing the number of preterm births and improving outcomes for mother and baby. Optimal route of delivery for preterm vertex neonates has been a controversial topic in the obstetric and neonatal community for decades and continues to be debated. We reviewed 22 studies, most of which have been published over the last five years with an aim to find answers to the clinical questions relevant to deciding the mode of delivery. Findings suggested that the neonatal outcome does not depend on the mode of delivery. Though Caesarean section rates are increasing for preterm births, it does not prevent neurodisability and cannot be recommended unless there are other obstetric indications to justify it. Therefore, clinical judgement of the obstetrician depending on the individual case still remains important in deciding the mode of delivery.

## 1. Introduction

Choosing the safest method of delivery and preventing preterm labour to reduce the number of preterm births and improve the outcomes for mother and baby is an obstetric challenge. 

Optimal route of delivery for preterm vertex neonates has been a controversial topic in the obstetric and neonatal community for decades and continues to be debated. The value of Caesarean section in preterm labour is less clear [1, 2]. This has not been subjected to robust randomised controlled trial. The present evidence comprises of a number of case series, systematic review of controlled trials with variable results, from beneficial through equivocal to no benefit. Despite the uncertainty regarding benefits for preterm vertex neonates, cesarean section delivery rates have increased.

The aim of this paper is to review the recent literature to assess the effect of the mode of delivery on preterm vertex neonates. We present a series of questions and aim to find answers based on recent evidence.

## 2. Background

Preterm birth refers to the birth of a baby at less than 37 weeks of gestational age. Preterm birth is the major cause of neonatal mortality in the developed countries. In the UK, infant mortality among preterm births was 42/1000 live births in 2005, compared with 5/1000 live births overall [3].

Premature infants are at greater risk for short- and long-term complications, including disabilities and impediments in growth and mental development. Most mortality and morbidity affects very preterm infants (those born before 32 weeks of gestation) and especially extremely preterm infants (those born before 28 weeks of gestation). Late preterm birth (32 to 36 + 6 weeks of gestation) is associated with less risk than very preterm birth, but there is a growing recognition that even in this group there is increased risk of infant death [3]. The risk of death or neurosensory disability increases with decreasing gestational age [4].

 The method of delivery is dependent on a variety of factors, and preterm labor per se does not dictate this one way or the other. Though the issue of how best to deliver is of importance to both obstetricians and neonatologists, there is little evidence from controlled studies on which to base the management of preterm birth. A Cochrane systematic review [2] commented that not enough studies have been done to provide adequate evidence.

Caesarean section has been postulated to have a theoretical advantage over vaginal delivery in premature infants. This benefit may be the result of the avoidance of prolonged labour, allowing a less traumatic birth [5]. On the other hand, preterm Caesarean section can be technically difficult and may require performing a classical Caesarean section with adverse risks like scar dehiscence in future pregnancy [6]. It also has other maternal risks associated with Caesarean section. Hence, vaginal birth is the preferred mode in the absence of other obstetric indications due to reduced maternal complications. However, it involves the risk of hypoxia and future neurodisability to the baby. Balancing the fetal versus maternal risks and safety continues to pose challenges.

Therefore, we present the review of recent evidence to find out whether the decision-making process between obstetricians and neonatologists, regarding mode of delivery, can be made easier or if the debate continues leaving it on the clinical judgement of the obstetrician.

## 3. Discussion

### 3.1. Does Mode of Delivery Influence Neonatal Outcome in Preterm Births?

New research suggests that the mode of delivery of very preterm infants whether vaginally or by Caesarean has little effect on neonatal outcomes. The findings come from a retrospective study unveiled at the American Congress of Obstetricians and Gynecologists 58th Annual Clinical Meeting in May 2010 [7]. This study included 126 preterm vertex singleton births with gestational age ranging from 23 to 30 weeks. The researchers compared outcomes that included neonatal deaths, intraventricular hemorrhage, necrotizing enterocolitis, respiratory distress syndrome, and clinical sepsis in 52 infants delivered by Caesarean and 74 delivered vaginally. Their conclusion was that mode of delivery does not provide any significant advantage in decreasing infant morbidity and mortality.

Similar findings came from a retrospective cohort study which was published recently [8]. They included all singleton deliveries occurring after spontaneous onset of labour between 25 + 0 and 32 + 6 weeks of gestation. 109 cases of spontaneous preterm labour were retrospectively selected, including 50 (45.8%) Caesarean sections and 59 (54.2%) vaginal deliveries. The neonatal outcomes compared between Caesarean and vaginal deliveries were perinatal death, cranial findings compatible with haemorrhage or white matter disease. The study concluded that in severely premature infants born after spontaneous onset of labour, the risk of adverse perinatal outcome does not seem to depend upon the mode of delivery. 

Another study [9] included 124 preterm babies of higher gestations ranging from 30 to 35 weeks and compared the outcomes of 70 neonates born vaginally and 54 neonates born by Caesarean. Neonatal mortality rate was 20 percent for infants in Caesarean group as compared to 10 percent for vaginal group. There was no significant difference in the neonatal morbidity among both the groups.

Different to the above studies which were based on gestational age is one study [10] that assessed the survival advantage of premature newborns according to the mode of delivery based on birth weights (500–999 g, 1000–1499 g, 1500–1999 g, and 2000–2499 g). Overall Caesarean delivery rate in this group was 32.2%. Among preterm newborns with birth weight 500–999 g, 68 children were delivered vaginally and 5 by Caesarean section (5.7% and 0.4% of all preterm babies, resp.). None of the infants survived. The percentage of children from Caesarean deliveries in the other groups was higher: for preterm infants with birth weight 1000–1499 g—3.2%, 1500–1999 g—8.8%, and 2000–2499 g—19.8%. A survival advantage associated with Caesarean section was observed in neonates with birth weight 1000–1499 g (*P* < 0.01). On the basis of this study, it was concluded that Caesarean delivery is associated with a decreased neonatal mortality risk in preterm neonates only in those with birth weight of 1000–1499 g. 

Based on the above studies, most agree that neonatal outcome does not depend upon the mode of delivery except one which found that Caesarean delivery could potentially reduce mortality in preterm neonates of birth weight of 1000–1499 g.

### 3.2. Does Caesarean Section Enhance the Survival Rate of Preterm Vertex Infants?

Given the continuing debate about the benefits of Caesarean section for very preterm infants, MOSAIC (models of organising access to intensive care for very preterm babies in europe) project sought to describe Caesarean section rates for infants between 28 and 31 weeks of gestation (*n* = 3310) in ten European regions and their association with regional mortality and short-term morbidity [11]. There were no regional level correlations between Caesarean section rates and mortality and morbidity. The conclusion was that, with the exception of pregnancies with hypertension and growth restriction, there was broad variation in very preterm Caesarean section rates between regions after adjustment for clinical factors. It was suggested that given the maternal risks associated with Caesarean section, more research on its optimal use for very preterm deliveries is necessary. 

Previous evidence comes from a Cochrane systematic review done in 2001 [2] which assessed the effects of a policy of elective Caesarean delivery versus selective Caesarean delivery (intention to deliver vaginally with recourse to Caesarean section) for women in preterm labour. Six randomized controlled trials with 122 women were included, and all trials reported recruiting difficulties. No significant differences between elective and selective policies for Caesarean delivery were found for fetal, neonatal, or maternal outcomes. Another publication of the same systematic review [1] found that odds of serious maternal morbidity were increased in the Caesarean section group (OR 6.2; 95% CI 1.3–30.1). 

#### 3.2.1. Evidence Supporting No Benefit of Caesarean Section on Survival Rate in Early and Very Low Birth Weight Preterm Births

An earlier published study [12] investigated the factors associated with Caesarean delivery and the relationship between mode of delivery and mortality in singleton vertex-presenting very low birth weight (< or = 1500 g) live born infants. 2955 singleton vertex-presenting very low birth weight infants born at 24–34 were included. The primary outcome measure was mortality defined as death prior to discharge. Caesarean delivery rate was 51.7%. Caesarean delivery was directly associated with increasing maternal age and gestational age, small for gestational age infants, maternal hypertensive disorders, and antepartum haemorrhage and was inversely related to premature labour and prolonged rupture of membranes. Mortality rate prior to discharge was lower after Caesarean delivery (13.2% versus 21.8%), but in the multivariate analysis, adjusting for the other risk factors associated with mortality, delivery mode had no effect on infant survival (OR 1.00, 95% CI 0.74–1.33). This study concluded that Caesarean delivery did not enhance survival of vertex-presenting singleton very low birth weight babies.

Two other recent studies support this finding. The first was a retrospective cohort study [13] undertaken to compare neonatal outcome by method of delivery in very low birth weight less than 1500 g vertex-presenting fetuses. 2466 very low birth weight singleton liveborn vertex-presenting fetuses at less than 28 weeks were included, and analyses were stratified by birth weight, gestational age, and growth restriction to assess subgroup differences. This study found that Caesarean delivery offered no survival advantage to very low birth weight infants when compared with vaginal delivery. Survival benefit was noted for growth-restricted infants although only 12% of such infants delivered vaginally. The second study [14] done by the coauthor was also a retrospective study to evaluate the obstetric management and perinatal outcome of extreme prematurity (22–27 weeks) over a period of one year. A total of 57 babies were included and Caesarean section was the mode of delivery in 32%. Only 12.5% of babies delivered by Caesarean section at less than 27 weeks survived as compared with 70% survival rate at 27 weeks. Therefore, no survival advantage was noted among the babies delivered by Caesarean section below 26 weeks.

#### 3.2.2. Evidence Supporting Benefit of Caesarean Section on Survival Rate of Early and Very Low Birth Weight Preterm Infants

Three studies provide evidence to support the above. First was a study [15] done in the United states which found that Caesarean section does seem to provide survival advantages for the most immature infants delivered at 22 to 25 weeks of gestation, independent of maternal risk factors for Caesarean section. Two other reports [16, 17] agreed to this finding and showed that survival advantage was associated with Caesarean delivery in the birth weight group of less than 1300 g.

Most of the above-mentioned studies included early preterm infants, but a study done in the United states [18] assessed the impact of Caesarean section on intermediate (32-33 weeks) and late (34–36 weeks) preterm births. The data suggested that for low-risk preterm infants at 32 to 36 weeks' gestation, independent of any reported risk factors, primary cesarean section may pose an increased risk of neonatal mortality and morbidity. 

Therefore, recent evidence regarding this question is conflicting. Three studies [12–14] suggest that Caesarean section does not enhance survival of vertex-presenting singleton very low birth weight babies and cannot be routinely recommended unless there are other obstetric indications. Other three [15–17] have found Caesarean delivery to be beneficial in infants less than 26 weeks of gestation or birth weight less than 1300 g. Consideration though should also be given to the technical difficulty associated with Caesarean sections at earlier gestations which can increase maternal morbidity.

### 3.3. Is Caesarean Section Beneficial for Preventing Future Neurodisability in Very Low Birth Infants?

Study from a district General Hospital in United Kingdom [19] included all infants weighing <1,250 g born between January 1995 and December 2003 and followed up at two years of age for assessment of the neurodevelopmental status by an independent paediatrician. 213 infants were analysed, of which 103 were born by vaginal delivery and 110 by Caesarean section They did not find any significant difference in the overall incidence of neurodisability in the infants born by Caesarean section as compared to those delivered vaginally. It was also noted that neurodisability was equally greater in babies with birth weight of 750 grams or less and/or born at 26 weeks or less gestation. Conclusion was that despite the increasing tendency to deliver extremely preterm babies by Caesarean, it was not associated with either reduced mortality or neurodisability at two years of age, and the method of delivery of very low birth weight premature infants should be based on obstetric or maternal indications rather than the perceived outcome of the baby. This is supported by another retrospective cohort study [20] which included a total of 1606 extremely low birth-weight infants (birth weight of 401–1000 g) who were born by cesarean delivery and 1273 could be followed up at 18 to 22 months of corrected age. They found that in extremely low birth-weight infants who were born by cesarean delivery, and after control for other risk factors, labor does not appear to play a significant role in adverse neonatal outcomes and neurodevelopmental impairment at 18 to 22 months of corrected age.

Similar findings came from another single-centre retrospective cohort study [21] of 84 cases of extremely low birth weight infants based on gestational age ( below 28 weeks of gestation, 40% at or less than 25 weeks) performed in Italy. This study evaluated the impact of mode of delivery and timing of Caesarean section in extremely preterm births on long-term survival and psychomotor outcomes. Mortality and survival with neurological disabilities at 18 months of life were considered outcome measures. They found that mode of delivery and labour seem not to play a significant role in adverse neonatal outcomes, either mortality or neurodevelopmental impairment, in extremely low birth weight infants. 

Two other studies [22, 23] investigated the association between the mode of delivery and intraventricular haemorrhage (IVH) in preterm infants. In the recent study [22] done which included infants with gestational age less than or equal to 28 weeks, Caesarean delivery was found to decrease the risk of developing IVH in extremely preterm infants including the most severe grades of IVH. This is contradictory to the finding from a previous study [23] which investigated the association between delivery mode and grade 3-4 intraventricular hemorrhage in singleton, vertex presenting, very low birth weight (1,500 g or less) liveborn infants. They found that odds for severe intraventricular hemorrhage were not influenced by the mode of delivery in vertex-presenting singleton very low birth infants after controlling for gestational age.

To summarize, recent evidence suggests that Caesarean section could decrease the risk of severe IVH in extremely preterm infants but does not reduce future neurodisability.

### 3.4. Is Vaginal Delivery Safe?

Vaginal delivery of preterm infant is associated with less maternal morbidity than caesarean section. The impact of vaginal delivery on 397 premature infants (44% born vaginally) weighing less than 1251 g was explored in one of the studies [24]. Outcomes measured were death, severe IVH, and periventricular leukomalacia (PVL). This study found that vaginal delivery was associated with higher risk of PVL. In infants weighing less than 751 g delivered vaginally, severe IVH is higher though the negative impact of vaginal delivery mode decreases as birth weight category increases.

Another study [25] which included 2,094 live births of infants at 23 + 0 to 27 + 6 of weeks gestation found that for preterm vertex without any other obstetric complications, 4 out of 5 infants were delivered vaginally without any increase in the risk.

Most of the other studies described in the earlier sections have not shown any difference in the neonatal outcome when vaginal and Caesarean deliveries were compared.

In summary, vaginal delivery can be associated with increased risk of IVH in very low birth weight infants, but no difference in the neonatal outcome and future neurodisability has been shown in most of the studies when compared to Caesarean section. Therefore it is reasonable to consider vaginal delivery safe in light of the accumulated evidence.

## 4. Conclusion

Effective care cannot be based on meta-analysis of well-designed controlled trials because none of the attempts have come to a conclusion. Recent findings from a number of studies have demonstrated that the neonatal outcome does not depend on the mode of delivery. Though Caesarean section rates are increasing for preterm births, there is conflicting evidence regarding its benefits in increasing the survival rate for early and very low birth weight preterm births. Moreover, it does not prevent neurodisability and cannot be recommended in light of recent evidence unless there are other obstetric indications to justify it. Future randomized controlled trials are necessary to support this recommendation which would be helpful in reducing the Caesarean section rates for preterm births.

It is important to consider the obstetric history, likely interval between induction and delivery in the context of deterioration of maternal health, probability of achieving a vaginal delivery compared to a risk of emergency section, presentation, and prelabour condition of the fetus. Hence, depending on the individual case, clinical judgment of the obstetrician still remains important in deciding the mode of delivery.
